# Social Media, Anxiety and COVID-19 Lockdown Measurement Compliance

**DOI:** 10.3390/ijerph20054416

**Published:** 2023-03-01

**Authors:** Stephanie Rodriguez-Besteiro, Ana Isabel Beltran-Velasco, José Francisco Tornero-Aguilera, Marina Begoña Martínez-González, Eduardo Navarro-Jiménez, Rodrigo Yáñez-Sepúlveda, Vicente Javier Clemente-Suárez

**Affiliations:** 1 Faculty of Sports Sciences, Universidad Europea de Madrid, Tajo Street, s/n, 28670 Madrid, Spain; 2 Facultad de Ciencias de la Vida y de la Naturaleza, Universidad Antonio de Nebrija, 28240 Madrid, Spain; 3 Department of Social Sciences, Universidad de la Costa, Barranquilla 080002, Colombia; 4 Facultad de Ciencias de la Salud, Grupo de Investigación en Medicina y Biotecnología—IMB, Universidad Libre seccional Barranquilla, Barranquilla 080001, Colombia; 5 Faculty of Education and Social Sciences, Universidad Andres Bello, Viña del Mar 2520000, Chile

**Keywords:** COVID-19, confinement, social media, lockdown, anxiety, risk perception

## Abstract

The aim of the present research was to analyze the effect of anxiety levels during the COVID-19 pandemic in the use of social media and compliance with lockdown measures during the confinement. A total of 1723 participants (32.1% males and 77.9% females; 32.6 ± 9.2 years) were interviewed by a Spanish version of the Spielberger State-Trait Anxiety Inventory. From the results obtained, the sample was divided into two 50th percentile groups, a high anxiety group (HAG) and a low anxiety group (LAG). We found how the LAG had lower use of social networks such as Facebook and Twitter during confinement. Also, this group presented a higher rate of leaving home during the confinement and higher values in people with whom they had lived with during confinement than high anxiety group. Regardless of the lack of results in the remaining variables, the present study nuances the high levels of anxiety experienced during COVID-19 confinement. The multifactorial analysis of factors related to the perception of anxiety during COVID-19 confinement may be a useful tool to measure multiple social behaviors when examining mental health factors. Thus, explaining and preventing the psychological consequences of the COVID-19 pandemic. The present knowledge could be used to determine key intervention factors for reducing the perception of fear and anxiety.

## 1. Introduction

In December 2019, a new coronavirus emerged in Wuhan (China) and soon spread around the world. To date, it has infected and killed millions of people, generating a significant health and economic crisis [1]. Since its inception, governments around the world have implemented extraordinary measures to reduce the spread of the virus. One of these was the total confinement of the population in their homes, disrupting most daily activities and causing separation from loved ones and loss of freedom. Consequently, a significant percentage of the population has experienced intense emotional adjustment reactions [2]. Therefore, it has been shown that the death of family members and increased social adversity can lead to adverse psychological effects, increasing the risk of emotional disorders, insomnia, low mood, irritability, depression and post-traumatic stress symptoms [3]. Numerous authors point out that the COVID-19 pandemic has impacted the mental health of people around the world, causing psychological and social distress [4,5].

Several studies have pointed out the effects of the COVID-19 pandemic and its consequences. As a result of the quarantines, it was observed that people’s anxiety levels increased, as well as the consumption of inflammatory foods, alcohol abuse, dependency symptoms and avoidance behaviors [6,7]. An increased risk of depression and post-traumatic symptoms was also found in patients with elevated levels of distress [8]. Overall, there are several factors that may increase or decrease the risk of developing symptoms of mental illness because of prolonged exposure to stressful stimuli, such as quarantines [9]. These measures established by the various states have been aimed at curbing the spread of COVID-19 and its collateral effects. The scientific community has begun to review the consequences that these measures have had and continue to have on people. Some authors explain that the spread of a disease can led to an increase in anxiety in the community, which can affect the prevention measures to be taken, such as changing people’s behavior and non-compliance with the measures imposed [10,11,12,13].

However, although the pandemic seems to be something that we are leaving behind as a society, in some countries it is still present, with major anti-viral measures and numerous confinements of the population. At the end of March 2022, the government of Shanghai, China’s financial capital, announced a strict lockdown that would affect all its inhabitants. These restrictions were to end weeks later; however, the increase in incidence has given them a prolonged uncertain future and uneven application by areas depending on the number of cases. During this period, many images have been shared on social networks showing the anxiety to which the population is being exposed, causing all these measures to be discussed and questioned [14,15,16].

Throughout the pandemic, all kinds of news was published around the world, both in the media and through social media. However, not all this news was true, often generating unnecessary unrest in society. This is what is known as fake news. The term ‘information’ from a journalistic point of view indicates that it must contain the truth, without the need for further adjectives [17]. However, nowadays, information does not only come from the traditional media but can come from anywhere, such as social networks, and filter into the collective knowledge as truth without being so [18]. The term “infodemic” was used to describe the wave of misinformation that was spread during the first COVID-19 outbreak [19]. A report conducted in the first week of the UK confinement found that 49% of people used social media to access news and information about COVID-19 and 46% reported seeing false or misleading information related to the pandemic [20]. COVID-19 has provided a huge opportunity for fake news to spread, sometimes causing damage to public safety by diffusion falsehoods about issues such as the origin of the virus, COVID-19 prevention and control procedures, the number of deaths and the harmful effects of vaccines [21]. Therefore, understanding the nature of fake news is quite complicated for several reasons. First, fake news is easily mixed with real news, making it difficult to distinguish the truth from the fake. On the other hand, a recent study has shown that fake news spreads faster than real news, quickly going viral and making it difficult to keep up with real news [22,23].

The failure to stop the spread of such fake news about the 2019 coronavirus disease (COVID-19) caused, especially at the beginning, panic, fear and chaos in society [24]. Some research indicates that information overload can lead to increased stressful situations, which can make it more likely to share fake news [25,26,27]. In addition, this exposure to infodemia can lead to increased panic, stress or more significant psychological disorders [28,29,30].

The COVID-19 pandemic changed the way people around the world use social media. Social media services were used to disseminate information and find humor and distraction from the pandemic through Internet memes [31]. However, social distancing forced a lifestyle change and put a lot of pressure on people’s mental health [32,33,34,35]. Even though there are currently some studies that analyze the effect of the pandemic on the consumption of social networks, there are not many that analyze how anxiety levels are related to the consumption of social networks. We hypothesized that people who had higher levels of anxiety during COVID-19 confinement will have been more compliant with the confinement and that that led to higher consumption of social networks. Therefore, the objective of the present research was to analyze the effect of anxiety levels during the COVID-19 pandemic on the use of social media and compliance with lockdown measures during the confinement.

## 2. Materials and Methods

We analyzed 1723 participants (32.1% males and 77.9% females; 32.6 ± 9.2 years). The participants in this research were of Colombian nationality, from the Atlantic region, who were contacted by email to request their participation. Data were collected during the first four months of the COVID-19 pandemic and analyzed in the following months. All the participants filled in an informed consent form following the Helsinki Declaration guidelines and all the procedures were approved by the University Ethics Committee (CIPI/18/074). A Spanish version of the Spielberger State-Trait Anxiety Inventory [36] was used. A reduced version composed of 6 items assessing anxiety and answered on a four-point Likert scale where 1 means not at all and 4 means very much. From the results obtained, the sample was divided into two groups, above the median, high anxiety group (HAG) and below the median, low anxiety group (LAG).

Also, the following parameters were analyzed:

*Behavioral descriptive:* Age (years), gender, weight (kg), quality of sleep and need for medication to sleep. Physical activity habits, among which the following were included: average steps per day in the last week, if any physical activity has been performed in the last 7 days, minutes of physical activity. Number of meals consumed the day before and motivation, measured with a VAS of 0–10. Motivation in a 0 to 100 VSA

*Use of social networks:* The use of social networks during confinement was measured with a scale in which 0 corresponded to low use of social networks and 10 to high use of social networks. The social networks analyzed were radio, TV, press, WhatsApp, Facebook, Instagram, Twitter, YouTube, local government social networks, national government social networks, influencers’ social networks and news social networks.

*COVID-19 compliance:* Perception of the dangerousness of the COVID-19 virus. The item is on a scale of 0 to 10. If relatives were in at-risk groups. Number of days in confinement. Number of times leaving home during confinement. Reason for leaving home during the confinement period. How many people have lived with you during the confinement? This was measured on a self-perception scale, indicating the number of people with whom the student lived. Loneliness during confinement, a Spanish version of the UCLA Loneliness Scale [37]. This scale measures loneliness. A condensed version consisting of three items answered by a three-point Likert scale, where 1 means never and 3 means frequently, was used in this research. Misses physical contact during confinement. Relationship with cohabitants during confinement. Artistic activity during confinement. Spiritual activity during confinement. Frequency of hand washing, the item is on a scale of 0 to 10. Frequency of mask use, the item is on a scale of 0 to 10. Frequency of use of hydro-alcoholic gel, the item is on a scale of 0 to 10. Frequency of use of social distance, the item is on a scale of 0 to 10. Frequency of avoidance of physical contact, the item is on a scale of 0 to 10. Frequency of disinfection of own objects, the item is on a scale of 0 to 10. Frequency of disinfection of food, the item is on a scale of 0 to 10. Frequency of use of products that strengthen the immune system, the item is on a scale of 0 to 10 (Table 1).

Statistical analyses were performed with the Statistical Package for the Social Sciences (SPSS) version 22.0 (SPSS Inc., Chicago, IL, USA). Descriptive statistics (mean and standard deviation) were calculated for each of the variables. Kolmogorov–Smirnov tests were performed to analyze the normality and homogeneity of each variable. An independent *t*-test was performed to analyze the differences between groups. The significance level was set at *p* ≤ 0.05. Finally, a multiple regression was conducted with the use of social networks and anxiety levels. The significance level was set at *p* ≤ 0.05.

## 3. Results

Data are presented as mean ± standard deviation. The high anxiety group (HAG) presented an average age of 31 years, whereas the low anxiety group (LAG) presented an average age of 34 years. This high anxiety group also presented a significantly higher number of women than men (*p*: 0.000). The low anxiety group presents a better sleep quality and motivation than the HAG (Table 2).

In relation to the use of social networks, significant differences were found between groups in the use of Facebook and Twitter, where the low anxiety group had a lower use of both networks than the high anxiety group. No significant differences between groups were found in the use of networks such as radio, TV, press, WhatsApp, Instagram, YouTube, local government social networks, national government social networks, influencers’ social networks and news social networks (Table 3).

In Table 4 is shown the results of the multiple regression of social networks and anxiety levels. The use of TV, Facebook, local government social networks and national government social networks presented the higher influence in anxiety levels.

According to differences between groups in COVID-19-related behaviors, significant differences were found. The low anxiety group presented a higher rate of leaving home during confinement than the high anxiety group. Likewise, the low anxiety group presented higher levels of missing physical contact during confinement than the high anxiety group. The low anxiety group presented higher values for people with whom they had lived with during confinement than the high anxiety group. No other significant differences were found in the rest of the variables analyzed corresponding to COVID-19-related behaviors between the groups (Table 5).

## 4. Discussion

The aim of the present research was to analyze the effect of anxiety levels during the COVID-19 pandemic in the use of social media and compliance with lockdown measures during the confinement. We hypothesized that people who have had higher levels of anxiety during COVID-19 confinement will have been more compliant with the confinement and that this will lead to higher consumption of social networks. Our hypothesis was compiled because the data indicate that participants belonging to the group with higher levels of anxiety have had a higher consumption of some social networks such as Facebook and Twitter during COVID-19 confinement. This high anxiety group has lived with fewer people during the confinement and has respected more COVID-19 sanitary measurements than those participants with lower anxiety levels.

The results obtained in this research support findings of previous research showing an association between age differences and anxiety levels, since the higher anxiety group is made up of younger participants than the low anxiety group [38,39]. The last decade has seen a change in the behavioral patterns of young adults, who are more connected to technology than in previous generations. This has different consequences, such as the fact that young adults today seem to have fewer social skills when it comes to behaving with their peers [40,41,42]. There is sufficient empirical evidence that indicates that social skills are related to the psychological wellbeing of people, especially adolescents [43]. Deficits in social skills have been related to various pathologies such as anxiety. Several studies indicate that the presence of social skills in young adults favors social adaptation and decreases the possibility of greater psychological inflexibility, which leads to higher levels of anxiety. This anxious symptomatology is often related to poor sleep quality. Young adults, particularly college students, are increasingly recognized as a population group that is greatly affected by poor sleep quality. One study found that anxiety predicted daytime sleepiness [44]. Similarly, more recent evidence suggests that insomnia or poor sleep quality is bidirectionally related to anxiety [45,46,47,48]. This is related to the findings obtained in the present research, where the high anxiety group presented lower levels of sleep quality than those participants who were part of the low anxiety group. Anxiety is also related to motivation levels in young adults. One study found a negative correlation between high levels of anxiety and achievement motivation [49]; therefore, and related to our results, young adults with higher levels of anxiety will have lower levels of motivation and lower achievement expectations. Although the results obtained in the present study do not have significant levels, anxiety levels can be related to the physical activity and exercise performed by people. Lindegard et al. studied 69 people (65% women) with anxiety who were advised to follow an 18-week physical training program. At the end of the program, they divided the participants into three groups: those who had not followed the program, those who had followed it partially and those who complied with it to a high degree. The results showed that the people in the latter two groups significantly decreased their anxiety levels [50,51].

In relation to COVID-19 compliance differences, subjects with a higher level of anxiety were those who left the house the least during confinement. According to the authors, this behavior is related to the perception of fear [52]. In this line, there are factors such as motivation [53] or sleep quality [54] that are closely related to the perception of fear during confinement [7]. Indeed, these factors are statistically significant in the group with the highest anxiety and also concordant with other studies in the same population group [55]. This opens new possible key intervention factors for reducing the perception of fear and anxiety.

Likewise, the subjects with the highest anxiety are those who most missed physical contact during confinement. In this line, a recent review shows similar results, emphasizing females, the young, the unemployed and those with previous mental health or psychiatric illnesses as the most vulnerable [56]. Authors suggest that this response related to the feeling of loneliness and is linked to the psychological profile, with greater psychological inflexibility and neurocentrism [57]. Furthermore, the group with the greatest anxiety is the one that has lived with the least number of people during confinement. This highlights the construct of group belonging. It appears that a sense of belonging is a useful concept pertinent to exploration of better social and psychological functioning [58]. The concept itself is related to loneliness, depression and anxiety. In this line, the authors found that greater levels of loneliness and isolation during quarantine were related to greater anxiety in university students [59], with greater incidence in women [7], children and adolescents [60]. These findings reveal the importance of considering multiple social behaviors when examining mental health factors.

Social media was one of main channels updating the COVID-19 information [61]. According to some research, people who were frequently exposed to social media had higher levels of anxiety as well as other pathologies such as depression [62]. To determine the principal sources of anxiety of the participants in the present research study, a regression analysis was carried out in which we found that the use of TV, Facebook, local government social networks and national government social networks presented the greatest influence on anxiety levels; this is likely due to the continuous presentation of news and content related to COVID-19 in the different networks [63]. Likewise, we found that people with higher anxiety values used more Twitter and Facebook social networks. The news posted on these social networks is usually not contrasted informative but is defined by the media or the person who publishes it [64]. This favors that a negative climate is generated in relation to a given topic and is fed back with comments on the original news or retweets [65,66]. This may explain why these two networks are the most used by people with higher levels of anxiety, but it may also mean that the continued use of these networks increases these levels of anxiety as the person is subjected to unverified information that can generate discomfort and uncertainty for consumers [67]. Although no significant results were obtained in the present research on other social networks, several studies indicate that there has been an increased use of social networks and messaging services such as WhatsApp during the COVID-19 pandemic [56,67]. This, in a pandemic scenario, in which the information provided by various national and international organizations, together with one’s own beliefs and knowledge, will cause a person with moderate or high levels of anxiety to feel a certain insecurity about what is happening. In addition, people who have higher levels of anxiety in their daily lives usually have a greater involvement in their interaction in social networks, seeking information but also a counterpart during the time they spend on this connection [68]. Therefore, in these people it can lead to greater irritability or feelings of frustration and nervousness if they do not get what they initially expected [69]. This, which may be part of the individual’s personality, will be increased in an environment that is not possible to control and in an unknown context such as a new disease that affects people globally [70,71].

In this line, people with high levels of anxiety may find the use of social networks a source of anxiety because there is practically no privacy in these environments [72]. These tools, which are initially intended to distract people and encourage communication and social contact, can become a place where there is too much information, data that have not been rigorously contrasted, personal opinions, negative comments and even discussions that go viral in minutes and that can generate concern, anxiety or anger when they spread through different channels [73,74,75,76]. However, it should also be considered that even in people who do not usually have high levels of stress, it can develop from the use of social networks precisely because of the difficulty of emotional regulation that can be found behind these media. The negative effects on mental health can even facilitate the appearance of mood disorders such as depression, lack of impulse control and anxiety disorders that will impact on the vital functions of the individual, increasing the perception of loneliness, affecting the regularization of circadian cycles, memory, attention and other physical pathologies such as muscle pain caused by stress [77]. The use of new technologies is an area in which we must address how certain digital dynamics affect consumers and whether the prolonged and extensive use of certain channels should be moderated to avoid mental health problems [78].

Regardless of the lack of results in the remaining variables, the present study nuances the high levels of anxiety experienced during COVID-19 confinement. Large-scale reviews spanning Europe, Asia and Oceania determine the prevalence of all forms of depression as 20%, anxiety as 35% and stress as 53% in the combined study population of 113,285 individuals. Indeed, when compared with other time periods, depression, anxiety, stress, sleep problems, motivation and psychological distress in general population prevalence was found to be higher during the COVID-19 pandemic [79]. The large impact of COVID-19 on the mental health of the population produced a psychological health risk for an increasing number of population [80,81,82,83]. This situation causes health systems to have to be prepared [84,85], which requires an adapted emergency legislation and greater control of contextual variables that can affect the citizen’s stress response [67,86,87,88,89,90] and that follows sustainable development goals [91].

## 5. Limitations and Future Research Guidelines

The multifactorial analysis of factors related to the perception of anxiety during COVID-19 confinement may be a useful tool to measure the multiple social behaviors when examining mental health factors, thus explaining and preventing the psychological consequences of the COVID-19 pandemic. Furthermore, using questionaries allows significant information to be collected in a short period of time. The present knowledge could be used to determine key intervention factors for reducing the perception of fear and anxiety.

However, the present research presents some limitations. The main one being the lack of biological samples related to the stress response (cortisol, adrenaline, alpha amylase, etc.). However, since this was an online questionnaire, no other methods of evaluation were possible. Future lines of research will address the difference in stress, anxiety and digital consumption, considering both gender and cultural differences. In addition, an attempt should be made to collect individual samples that allow the analysis of the stress response.

Finally, it seems complex to understand whether the use of social networks is a trigger or a consequence of the symptomatology associated with mood disorders, so this is an interesting line of work to be addressed in future research.

## 6. Conclusions

As discussed, the COVID-19 pandemic disrupted people’s lives, especially during lockdown periods, leading to a change in their consumption of social networks. Based on the results we have achieved, we can affirm that people who had higher levels of anxiety during COVID-19 confinement were more compliant with the confinement and that this may have led to greater consumption of social networks. The results indicate that the high anxiety group (HAG) had a lower mean age than the low anxiety group (LAG). This high anxiety group also had a significantly higher number of women than men. Relating anxiety to social network consumption during quarantine, the low anxiety group had a lower use of Facebook and Twitter than the high anxiety group. Therefore, it can be said that when anxiety levels are lower, the person may not have as much need to consult these social networks. No significant differences between groups have been found in the rest of the social networks analyzed. In addition, relating anxiety to COVID-19 compliance, the low anxiety group presented a higher rate of leaving home during confinement than the high anxiety group. In the same way that this group presented higher levels of missing physical contact during confinement and higher values for people with whom they had lived with during confinement than the high anxiety group. Therefore, we can conclude that the higher the level of anxiety, the higher the consumption of social networks and the greater the commitment to comply with the compliance measures established in the COVID-19 pandemic.

## Figures and Tables

**Table 1:** Summary of measures analyzed.

Level of Anxiety	Behavioral Descriptive	Motivation	Use of Social Networks	COVID-19 Compliance
State-Trait Anxiety Inventory	Age	VAS of 0–100	Radio	A Spanish version of the UCLA Loneliness Scale
	Gender		TV	Perception of the dangerousness of the COVID-19 virus
	Weight		Press	Number of days in confinement
	Quality of sleep		Whastapp	Number of times leaving home during confinement
	Need for medication to sleep		Facebook	Reason for leaving home during the confinement period
	Physical activity in the last 7 days		Instagram	How many people have lived with you during the confinement?
	Number of meals consumed the day		Twitter	Loneliness during confinement
			Youtube	Relationship with cohabitants during confinement
			Local government social networks	Artistic activity during confinement
			National government social networks	Spiritual activity during confinement
			Influencers’ social networks	Frequency of hand washing
			News social networks	Frequency of use of hydro-alcoholic gel
				Frequency of avoidance of physical contact
				Frequency of disinfection of own objects
				Frequency of disinfection of food
				Frequency of use of products that strengthen the immune system

**Table 2:** Behavioral descriptive.

					95% Confidence Interval of the Difference	
	Low Anxiety Group (LAG)	High Anxiety Group (HAG)	*t*	*p*	Lower	Upper
Age (years)	34.43 ± 12.501	31.68 ± 11.221	4.643	0.000	1.589	3.914
Weight (kg)	71.07 ± 17.27	70.21 ± 28.59	0.677	0.498	−1.62547	3.33944
Quality of your sleep	80.94 ± 18.282	65.67 ± 22.731	14.835	0.000	13.247	17.284
Need medication to sleep	2.17 ± 3.968	1.90 ± 1.524	1.509	0.131	–0.083	0.639
Average steps per day in the last week?	1856.41 ± 2724.33	2123.76 ± 5470.69	–0.911	0.362	−843.164	308.465
Did you do any physical activity in the last 7 days?	1.47 ± 0.500	1.52 ± 0.500	−1.676	0.094	–0.091	0.007
Minutes of physical activity	188.33 ± 243.566	187.88 ± 318.226	0.023	0.982	−38.432	39.330
How many meals did you take the day before	5.27 ± 14.491	5.39 ± 17.886	–0.149	0.881	−1.750	1.502
Motivation	81.12 ± 54.794	60.34 ± 21.541	9.914	0.000	16.668	24.890

**Table 3:** Use of social networks between groups.

					95% Confidence Interval of the Difference	
	Low Anxiety Group (LAG)	High Anxiety Group (HAG)	*t*	*p*	Lower	Upper
Radio (0–10)	4.50 ± 3.146	4.42 ± 3.376	0.361	0.718	–0.326	0.474
TV (0–10)	4.52 ± 3.220	4.30 ± 3.295	1.273	0.203	−0.120	0.564
Press (0–10)	3.99 ± 3.292	3.81 ± 3.515	0.986	0.324	−0.178	0.537
Whatsapp (0–10)	3.72 ± 3.625	3.81 ± 3.645	−0.492	0.623	−0.482	0.289
Facebook (0–10)	2.67 ± 2.864	3.21 ± 3.236	−3.294	0.001	−0.860	−0.218
Instagram (0–10)	2.10 ± 2.323	2.09 ± 2.595	0.111	0.911	−0.245	0.274
Twitter (0–10)	2.06 ± 2.512	2.42 ± 2.671	−2.116	0.035	−0.677	−0.025
Youtube (0–10)	2.24 ± 2.606	2.32 ± 2.483	−0.540	0.589	−0.341	0.194
Local government social networks (0–10)	5.66 ± 3.267	5.97 ± 3.074	−1.787	0.074	−0.658	0.031
National government social networks (0–10)	5.97 ± 3.368	6.03 ± 3.310	−0.290	0.772	−0.416	0.309
Influencers’ social networks (0–10)	2.25 ± 2.985	2.24 ± 2.893	0.063	0.949	−0.308	0.329
News social networks (0–10)	2.69 ± 3.429	4.71 ± 3.372	−0.062	0.951	−0.381	0.358

**Table 4:** Multiple regression: impact of social networks on anxiety levels.

Source of Anxiety	Unstandarized Regression Coefficient	*t*	*p*
Radio (0–10)	0.008	0.140	0.889
Tv (0–10)	−0.134	−2.313	0.021
Press (0–10)	−0.054	−0.892	0.372
Whatsapp (0–10)	−0.053	−1.058	0.290
Facebook (0–10)	0.174	3.265	0.001
Instagram (0–10)	−0.149	−1.857	0.064
Twiter (0–10)	0.102	1.788	0.074
Youtube (0–10)	0.005	0.071	0.943
Local government social networks (0–10)	0.219	3.659	0.000
National government social networks (0–10)	−0.135	−2.606	0.009
Influencers’ social networks (0–10)	−0.031	−0.550	0.582
News social networks (0–10)	−0.008	−0.164	0.870

Multiple R = 0.221, Multiple R-squared = 0.049.

**Table 5:** Differences between groups in COVID-19-related behaviors.

					95% Confidence Interval of the Difference	
	Low Anxiety Group (LAG)	High Anxiety Group (HAG)	*t*	*p*	Lower	Upper
Level of perceived danger in the COVID-19 virus (0–10)	8.00 ± 7.396	8.26 ± 2.986	−0.903	0.367	−0.806	0.298
Relatives in risk groups	1.68 ± 2.401	1.57 ± 2.070	0.953	0.341	−0.117	0.338
Days in confinement	26.64 ± 18.724	27.38 ± 18.260	−0.782	0.434	−2.579	1.109
Leaves home during confinement	1.39 ± 3.226	1.13 ± 0.340	2.274	0.023	0.036	0.487
Reason for leaving home during the confinement period	1.16 ± 0.618	1.25 ± 1.04	−1.748	0.081	−0.176	0.010
Loneliness during confinement	1.81 ± 0.466	1.88 ± 2.662	−0.726	0.468	−0.269	0.124
Misses physical contact during confinement	1.93 ± 3.241	1.52 ± 2.043	2.843	0.005	0.125	0.682
Relationship with cohabitants during confinement	7.68 ± 3.299	7.75 ± 6.434	−0.274	0.784	−0.608	0.459
How many people have you lived with in confinement?	1.37 ± 9.660	1.19 ± 7.387	3.986	0.000	0.922	2.709
Artistic activity during confinement	1.82 ± 1.734	2.14 ± 4.480	−1.782	0.075	−0.668	0.032
Learning during confinement	1.36 ± 0.479	4.72 ± 57.822	−1.574	0.116	−7.552	0.827
Spiritual activity during confinement	1.48 ± 0.801	1.46 ± 0.661	0.454	0.650	−0.059	0.094
Frequency of hand washing (0–10)	6.67 ± 3.607	6.68 ± 3.560	−0.043	0.966	−0.422	0.404
Frequency of mask use (0–10)	5.01 ± 3.880	4.78 ± 3.758	1.016	0.310	−0.212	0.668
Frequency of use of hydro-alcoholic gel (0–10)	5.41 ± 3.729	5.49 ± 3.736	−0.341	0.734	−0.537	0.379
Frequency of use of social distance (0–10)	6.37 ± 3.878	6.28 ± 3.721	0.373	0.709	−0.355	0.521
Frequency of avoidance of physical contact (0–10)	7.18 ± 3.304	7.47 ± 3.033	−1.546	0.122	−0.654	0.078
Frequency of disinfection of own objects (0–10)	6.37 ± 3.591	6.67 ± 3.442	−1.465	0.143	−0.709	0.103
Frequency of disinfection of food (0–10)	5.55 ± 4.134	5.58 ± 4.151	−0.119	0.905	−0.507	0.449
Frequency of use of products that strengthen the immune system (0–10)	5.30 ± 3.670	5.09 ± 3.718	0.996	0.319	−0.211	0.648

## Data Availability

All data are presented in the manuscript.

## References

[1] W.H.O (2021). Coronavirus Disease (COVID-19) Pandemic. World Health Organization Coronavirus Disease (COVID-19) Pandemic. https://www.who.int/emergencies/diseases/novel-coronavirus-2019.

[2] Li S., Wang Y., Xue J., Zhao N., Zhu T. (2020). The Impact of COVID-19 Epidemic declaration on psychological consequences: A study on active weibo users. Int. J. Environ. Res. Public Health.

[3] Dugas M.J., Marchand A., Ladouceur R. (2005). Further validation of a cognitive-behavioral model of generalized anxiety disorder: Diagnostic and symptom specificity. J. Anxiety Disord..

[4] Zhang J., Lu H., Zeng H., Zhang S., Du Q., Jiang T., Du B. (2020). The differential psychological distress of populations affected by the COVID-19 pandemic. Brain Behav. Immun..

[5] Martín-Rodríguez A., Tornero-Aguilera J.F., López-Pérez P.J., Clemente-Suárez V.J. (2022). Dietary patterns of adolescent students during the COVID-19 pandemic lockdown. Physiol. Behav..

[6] Brooks S.K., Webster R.K., Smith L.E., Woodland L., Wessely S., Greenberg N., Rubin G.J. (2020). The psychological impact of quarantine and how to reduce it: Rapid review of the evidence. Lancet.

[7] Rodriguez-Besteiro S., Tornero-Aguilera J.F., Fernández-Lucas J., Clemente-Suárez V.J. (2021). Gender differences in the COVID-19 pandemic risk perception, psychology and behaviors of spanish university students. Int. J. Environ. Res. Public Health.

[8] Kim H.-C., Yoo S.-Y., Lee B.-H., Lee S.H., Shin H.-S. (2018). Psychiatric findings in suspected and confirmed middle east respiratory syndrome patients quarantined in hospital. Psychiatry Investig..

[9] Zdin S., Bayrak Özdin Ş. (2020). Levels and predictors of anxiety, depression and health anxiety during COVID-19 pandemic in Turkish society: The importance of gender. Int. J. Soc. Psychiatry.

[10] Rubin G.J., Wessely S. (2020). The psychological effects of quarantining a city. BMJ.

[11] Lima C.K.T., Carvalho P.M.D.M., Lima I.D.A.A.S., Nunes J.V.A.D.O., Saraiva J.S., de Souza R.I., da Silva C.G.L., Neto M.L.R. (2020). The emotional impact of Coronavirus 2019-nCoV (new Coronavirus disease). Psychiatry Res..

[12] Prieto-Molinaria D.E., Bravo A., Gianella L., de Pierola I., Luna Victoria-de Bona G., Merea Silva L.A., Lazarte Nuñez C.S., Uribe-Bravo K., Zegarra Á.C. (2020). Depresión y ansiedad durante el aislamiento obligatorio por el COVID-19 en Lima Metropolitana. Liberabit.

[13] Zheng L., Miao M., Lim J., Li M., Nie S., Zhang X. (2020). Is lockdown bad for social anxiety in COVID-19 regions?: A national study in the sor perspective. Int. J. Environ. Res. Public Health.

[14] Dyer O. (2022). COVID-19: Lockdowns spread in China as omicron tests “zero covid” strategy. BMJ.

[15] Shanghái, la Ciudad Cerrada por Covid: Escenas de un Confinamiento Radical. El País. https://elpais.com/videos/2022-04-16/shanghai-ciudad-cerrada-escenas-de-un-confinamiento-radical.html.

[16] Nieto A., Dea la Rúa Á. ¿Cómo se Vive en Shanghái el Confinamiento Masivo por Covid-19? CNN. https://cnnespanol.cnn.com/video/shanghai-covid19-confinamiento-masivo-experiencia-pkg-david-culver-redaccion-mexico/.

[17] Brajnovic L. (1979). El Ámbito Científico de la Información.

[18] Román-San-Miguel A., Valenzuela N.S.G., Zambrano R.E. (2020). Las fake news durante el estado de alarma por COVID-19. Análisis desde el punto de vista político en la prensa española. Rev. Lat. De Comun. Soc..

[19] Zarocostas J. (2020). How to fight an infodemic. Lancet.

[20] Ofcom (2020). COVID-19 News and Information: Consumption and Attitudes. Results from Week One of Ofcom’s Online Survey. https://www.ofcom.org.uk/research-and-data/tv-radio-and-on-demand/news-media/coronavirus-news-consumption-attitudes-behaviour/previous-results.

[21] Ball P., Maxmen A. (2020). The epic battle against coronavirus misinformation and conspiracy theories. Nature.

[22] Vosoughi S., Roy D., Aral S. (2018). The spread of true and false news online. Science.

[23] Gupta A., Li H., Farnoush A., Jiang W.K. (2021). Understanding patterns of COVID infodemic: A systematic and pragmatic approach to curb fake news. J. Bus. Res..

[24] Singh L., Bansal S., Bode L., Budak C., Chi G., Kawintiranon K., Padden C., Vanarsdall R., Vraga E., Wang Y. (2020). A first look at COVID-19 information and misinformation sharing on Twitter. arXiv.

[25] Islam A.K.M.N., Laato S., Talukder S., Sutinen E. (2020). Misinformation sharing and social media fatigue during COVID-19: An affordance and cognitive load perspective. Technol. Forecast. Soc. Chang..

[26] Laato S., Islam A.N., Farooq A., Dhir A. (2020). Unusual purchasing behavior during the early stages of the COVID-19 pandemic: The stimulus-organism-response approach. J. Retail. Consum. Serv..

[27] Laato S., Islam A.K.M.N., Islam M.N., Whelan E. (2020). What drives unverified information sharing and cyberchondria during the COVID-19 pandemic?. Eur. J. Inf. Syst..

[28] Mukhtar S. (2020). Psychological health during the coronavirus disease 2019 pandemic outbreak. Int. J. Soc. Psychiatry.

[29] Teng Z., Wei Z., Qiu Y., Tan Y., Chen J., Tang H., Wu H., Wu R., Huang J. (2020). Psychological status and fatigue of frontline staff two months after the COVID-19 pandemic outbreak in China: A cross-sectional study. J. Affect. Disord..

[30] Lelisho M.E., Pandey D., Alemu B.D., Pandey B.K., Tareke S.A. (2022). The negative impact of social media during COVID-19 pandemic. Trends Psychol..

[31] Nisar S., Shafiq M. (2018). Framework for efficient utilisation of social media in Pakistan’s healthcare sector. Technol. Soc..

[32] Coroiu A., Moran C., Campbell T., Geller A.C. (2020). Barriers and facilitators of adherence to social distancing recommendations during COVID-19 among a large international sample of adults. PLoS ONE.

[33] Hwang T.-J., Rabheru K., Peisah C., Reichman W., Ikeda M. (2020). Loneliness and social isolation during the COVID-19 pandemic. Int. Psychogeriatr..

[34] Son C., Hegde S., Smith A., Wang X., Sasangohar F. (2020). Effects of COVID-19 on college students’ mental health in the united states: Interview survey study. J. Med. Internet Res..

[35] De Vos J. (2020). The effect of COVID-19 and subsequent social distancing on travel behavior. Transp. Res. Interdiscip. Perspect..

[36] Van Knippenberg F., Duivenvoorden H., Bonke B., Passchier J. (1990). Shortening the state-trait anxiety inventory. J. Clin. Epidemiol..

[37] Russell D. (1996). UCLA Loneliness scale (Version 3): Reliability, validity, and factor structure. J. Pers. Assess..

[38] Silk J.S., Davis S., McMakin D.L., Dahl R.E., Forbes E.E. (2012). Why do anxious children become depressed teenagers? The role of social evaluative threat and reward processing. Psychol. Med..

[39] Villanueva A.R.E., Mamani R.P.P., Condori C.R.C., Saico C.R.Y. (2020). Habilidades sociales en adolescentes y funcionalidad familiar. Comuni@cción.

[40] Córdova Lalangui J.M. (2021). Estudio del Uso Problemático del Celular y su Relación con Flexibilidad e Inflexibilidad Psicológica en Jóvenes Universitarios de 18 a 25 años de la Ciudad de Loja Durante el Año 2021. Bachelor’s Thesis.

[41] Torrente E., Piqueras A., Orgilés M., Espada J.P. (2014). Asociación de la adicción a Internet con la ansiedad social y la falta de habilidades sociales en adolescentes españoles. Ter. Psicológica.

[42] Semrud-Clikerman M. (2007). Social Competent in Children.

[43] Lacunza A.B. (2010). Las habilidades sociales como recursos para el desarrollo de fortalezas en la infancia. Psicodebate.

[44] Hasler G., Buysse D.J., Gamma A., Ajdacic V., Eich D., Rössler W., Angst J. (2005). Excessive daytime sleepiness in young adults: A 20-year prospective community study. J. Clin. Psychiatry.

[45] Kim J.-M., Stewart R., Kim S.-W., Yang S.-J., Shin I., Yoon J.-S. (2009). Insomnia, depression, and physical disorders in late life: A 2-year Longitudinal Community Study in Koreans. Sleep.

[46] Johansson M., Jansson-Fröjmark M., Norell-Clarke A., Linton S.J. (2021). Changes in insomnia as a risk factor for the incidence and persistence of anxiety and depression: A longitudinal community study. Sleep Sci. Pract..

[47] Morphy H., Dunn K.M., Lewis M., Boardman H.F., Croft P.R. (2007). Epidemiology of insomnia: A longitudinal study in a UK population. Sleep.

[48] Alvaro P.K., Roberts R., Harris J.K. (2013). A systematic review assessing bidirectionality between sleep disturbances, anxiety, and depression. Sleep.

[49] De Tena P.A., Fernández R.M., Alberola S.P., González C.R. (2013). Ansiedad y motivación de logro en estudiantes universitarios: Un estudio correlacional. ReiDoCrea Rev. Electrónica Investig. Docencia Creat..

[50] Herrera Gutiérrez E., Brocal Pérez D., Mármol D.J., Rodríguez Dorantes J.M. (2012). Relación entre actividad física, depresión y ansiedad en adolescentes. Cuad. Psicol. Deporte.

[51] Guerra Santiesteban J.R., Gutiérrez Cruz M., Zavala Plaza M., Singre Álvarez J., Goosdenovich Campoverde D., Romero Frómeta E. (2017). Relación entre ansiedad y ejercicio físico. Rev. Cuba. Investig. Biomédicas.

[52] Jovančević A., Milićević N. (2020). Optimism-pessimism, conspiracy theories and general trust as factors contributing to COVID-19 related behavior—A cross-cultural study. Personal. Individ. Differ..

[53] Kim J., Yang K., Min J., White B. (2022). Hope, fear, and consumer behavioral change amid COVID-19: Application of protection motivation theory. Int. J. Consum. Stud..

[54] Siddique R.F., Ahmed O., Hossain K.N. (2021). Relationship between the fear of COVID-19 disease and sleep quality: The mediating role of stress. Heliyon.

[55] Gómez-Salgado J., Allande-Cussó R., Domínguez-Salas S., García-Iglesias J., Coronado-Vázquez V., Ruiz-Frutos C. (2021). Design of fear and anxiety of COVID-19 assessment tool in spanish adult population. Brain Sci..

[56] Rodríguez-Fernández P., González-Santos J., Santamaría-Peláez M., Soto-Cámara R., Sánchez-González E., González-Bernal J. (2021). Psychological effects of home confinement and social distancing derived from COVID-19 in the general population—A systematic review. Int. J. Environ. Res. Public Health.

[57] Martín-Rodríguez A., Tornero-Aguilera J.F., López-Pérez P.J., Clemente-Suárez V.J. (2021). The effect of loneliness in psychological and behavioral profile among high school students in Spain. Sustainability.

[58] Hagerty B.M., Williams R.A., Coyne J.C., Early M.R. (1996). Sense of belonging and indicators of social and psychological functioning. Arch. Psychiatr. Nurs..

[59] Dingle G.A., Han R., Carlyle M. (2022). Loneliness, belonging, and mental health in Australian university students pre-and post-covid-19. Behav. Chang..

[60] Loades M.E., Chatburn E., Higson-Sweeney N., Reynolds S., Shafran R., Brigden A., Linney C., McManus M.N., Borwick C., Crawley E. (2020). Rapid systematic review: The impact of social isolation and loneliness on the mental health of children and adolescents in the context of COVID-19. J. Am. Acad. Child Adolesc. Psychiatry.

[61] Bao Y., Sun Y., Meng S., Shi J., Lu L. (2020). 2019-nCoV epidemic: Address mental health care to empower society. Lancet.

[62] Gao J., Zheng P., Jia Y., Chen H., Mao Y., Chen S., Wang Y., Fu H., Dai J. (2020). Mental health problems and social media exposure during COVID-19 outbreak. PLoS ONE.

[63] Neria Y., Sullivan G.M. (2011). Understanding the mental health effects of indirect exposure to mass trauma through the media. JAMA.

[64] Marzouki Y., Aldossari F.S., Veltri G.A. (2021). Understanding the buffering effect of social media use on anxiety during the COVID-19 pandemic lockdown. Humanit. Soc. Sci. Commun..

[65] Wiederhold B.K. (2020). Using Social media to our advantage: Alleviating anxiety during a pandemic. Cyberpsychol. Behav. Soc. Netw..

[66] Levaot Y., Greene T., Palgi Y. (2020). The associations between media use, peritraumatic distress, anxiety and resilience during the COVID-19 pandemic. J. Psychiatr. Res..

[67] Clemente-Suárez V.J., Navarro-Jiménez E., Simón-Sanjurjo J.A., Beltran-Velasco A.I., Laborde-Cárdenas C.C., Benitez-Agudelo J.C., Bustamante-Sánchez Á., Tornero-Aguilera J.F. (2022). Mis–dis information in COVID-19 health crisis: A Narrative review. Int. J. Environ. Res. Public Health.

[68] Ekasari R., Widiastuty L., Wijaya D.R., Arranury Z., Karini T.A., Ahmad A. (2022). Level of anxiety among college students during COVID-19 pandemic. Interest J. Ilmu Kesehat..

[69] Sewall C.J., Goldstein T.R., Rosen D. (2021). Objectively measured digital technology use during the COVID-19 pandemic: Impact on depression, anxiety, and suicidal ideation among young adults. J. Affect. Disord..

[70] Bailey E., Boland A., Bell I., Nicholas J., La Sala L., Robinson J. (2022). The mental health and social media use of young australians during the COVID-19 pandemic. Int. J. Environ. Res. Public Health.

[71] Wang Q., Xie L., Song B., Di J., Wang L., Mo P.K.-H. (2022). Effects of social media use for health information on COVID-19–related risk perceptions and mental health during pregnancy: Web-based survey. JMIR Public Health Surveill..

[72] Basheti I.A., Mhaidat Q.N., Mhaidat H.N. (2021). Prevalence of anxiety and depression during COVID-19 pandemic among healthcare students in Jordan and its effect on their learning process: A national survey. PLoS ONE.

[73] Brailovskaia J., Margraf J. (2021). The relationship between burden caused by coronavirus (COVID-19), addictive social media use, sense of control and anxiety. Comput. Hum. Behav..

[74] Nekliudov N.A., Blyuss O., Cheung K.Y., Petrou L., Genuneit J., Sushentsev N., Levadnaya A., Comberiati P., Warner J.O., Tudor-Williams G. (2020). Excessive media consumption about COVID-19 is associated with increased state anxiety: Outcomes of a large online survey in Russia. J. Med. Internet Res..

[75] Güldal Ş., Kılıçoğlu N.A., Kasapoğlu F. (2022). Psychological flexibility, coronavirus anxiety, humor and social media addiction during COVID-19 pandemic in Turkey. Int. J. Adv. Couns..

[76] Pramukti I., Strong C., Sitthimongkol Y., Setiawan A., Pandin M.G.R., Yen C.-F., Lin C.-Y., Griffiths M.D., Ko N.-Y. (2020). Anxiety and suicidal thoughts during the COVID-19 pandemic: Cross-Country comparative study among Indonesian, Taiwanese, and Thai university students. J. Med. Internet Res..

[77] Chen Y., Wu J., Ma J., Zhu H., Li W., Gan Y. (2021). The mediating effect of media usage on the relationship between anxiety/fear and physician–patient trust during the COVID-19 pandemic. Psychol. Health.

[78] Goodwin R., Wiwattanapantuwong J., Tuicomepee A., Suttiwan P., Watakakosol R., Ben-Ezra M. (2021). Anxiety, perceived control and pandemic behaviour in Thailand during COVID-19: Results from a national survey. J. Psychiatr. Res..

[79] Lakhan R., Agrawal A., Sharma M. (2020). Prevalence of depression, anxiety, and stress during COVID-19 pandemic. J. Neurosci. Rural. Pract..

[80] Clemente-Suárez V.J., Dalamitros A.A., Beltran-Velasco A.I., Mielgo-Ayuso J., Tornero-Aguilera J.F. (2020). Social and psychophysiological consequences of the COVID-19 pandemic: An extensive literature review. Front. Psychol..

[81] Clemente-Suárez V.J., Fuentes-García J.P., Marcos R.D.L.V., Patiño M.J.M. (2020). Modulators of the personal and professional threat perception of olympic athletes in the actual COVID-19 crisis. Front. Psychol..

[82] Clemente-Suárez V., Navarro-Jiménez E., Jimenez M., Hormeño-Holgado A., Martinez-Gonzalez M., Benitez-Agudelo J., Perez-Palencia N., Laborde-Cárdenas C., Tornero-Aguilera J. (2021). Impact of COVID-19 pandemic in public mental health: An extensive narrative review. Sustainability.

[83] Clemente-Suárez V.J., Martínez-González M.B., Benitez-Agudelo J.C., Navarro-Jiménez E., Beltran-Velasco A.I., Ruisoto P., Arroyo E.D., Laborde-Cárdenas C.C., Tornero-Aguilera J.F. (2021). The impact of the COVID-19 pandemic on mental disorders. A critical review. Int. J. Environ. Res. Public Health.

[84] Soto-Baño M.A., Clemente-Suárez V.J. (2021). Psicología de emergencias en España: Análisis actual, normativa y proposición reguladora. Pap. Psicólogo.

[85] Soto-Baño M.A., Clemente-Suárez V.J. (2021). Psicología de emergencias en España: Delimitación conceptual, ámbitos de actuación y propuesta de un sistema asistencial. Pap. Psicólogo.

[86] Martínez-González M., Turizo-Palencia Y., Arenas-Rivera C., Acuña-Rodríguez M., Gómez-López Y., Clemente-Suárez V. (2021). Gender, anxiety, and legitimation of violence in adolescents facing simulated physical aggression at school. Brain Sci..

[87] Rodriguez-Besteiro S., Valencia-Zapata G., de la Rosa E.B., Clemente-Suárez V.J. (2022). Food consumption and COVID-19 risk perception of university students. Sustainability.

[88] Clemente-Suárez V., Navarro-Jiménez E., Moreno-Luna L., Saavedra-Serrano M., Jimenez M., Simón J., Tornero-Aguilera J. (2021). The impact of the COVID-19 pandemic on social, health, and economy. Sustainability.

[89] Clemente-Suárez V.J., Bustamante-Sanchez Á., Tornero-Aguilera J.F., Ruisoto P., Mielgo-Ayuso J. (2022). Inflammation in COVID-19 and the effects of non-pharmacological interventions during the pandemic: A review. Int. J. Mol. Sci..

[90] Bermejo-Franco A., Sánchez-Sánchez J.L., Gaviña-Barroso M.I., Atienza-Carbonell B., Balanzá-Martínez V., Clemente-Suárez V.J. (2022). Gender differences in psychological stress factors of physical therapy degree students in the COVID-19 pandemic: A cross-sectional study. Int. J. Environ. Res. Public Health.

[91] Clemente-Suárez V.J., Rodriguez-Besteiro S., Cabello-Eras J.J., Bustamante-Sanchez A., Navarro-Jiménez E., Donoso-Gonzalez M., Beltrán-Velasco A.I., Tornero-Aguilera J.F. (2022). Sustainable development goals in the COVID-19 pandemic: A narrative review. Sustainability.

